# Worldwide Protein Data Bank (wwPDB): A virtual treasure for research in biotechnology

**DOI:** 10.1556/1886.2021.00020

**Published:** 2021-12-15

**Authors:** PAYAM BEHZADI, MÁRIÓ GAJDÁCS

**Affiliations:** 1 Department of Microbiology, College of Basic Sciences, Shahr-e-Qods Branch, Islamic Azad University, Tehran, 37541-374, Iran; 2 Department of Oral Biology and Experimental Dental Research, Faculty of Dentistry, University of Szeged, 6720, Szeged, Hungary

**Keywords:** PDB, proteins, nucleic acids, RNA, DNA, drug design, vaccines, biotechnology, COVID-19

## Abstract

The Research Collaboratory for Structural Bioinformatics Protein Data Bank (RSCB PDB) provides a wide range of digital data regarding biology and biomedicine. This huge internet resource involves a wide range of important biological data, obtained from experiments around the globe by different scientists. The Worldwide Protein Data Bank (wwPDB) represents a brilliant collection of 3D structure data associated with important and vital biomolecules including nucleic acids (RNAs and DNAs) and proteins. Moreover, this database accumulates knowledge regarding function and evolution of biomacromolecules which supports different disciplines such as biotechnology. 3D structure, functional characteristics and phylogenetic properties of biomacromolecules give a deep understanding of the biomolecules’ characteristics. An important advantage of the wwPDB database is the data updating time, which is done every week. This updating process helps users to have the newest data and information for their projects. The data and information in wwPDB can be a great support to have an accurate imagination and illustrations of the biomacromolecules in biotechnology. As demonstrated by the SARS-CoV-2 pandemic, rapidly reliable and accessible biological data for microbiology, immunology, vaccinology, and drug development are critical to address many healthcare-related challenges that are facing humanity. The aim of this paper is to introduce the readers to wwPDB, and to highlight the importance of this database in biotechnology, with the expectation that the number of scientists interested in the utilization of Protein Data Bank’s resources will increase substantially in the coming years.

## INTRODUCTION

**Fig. 1.**
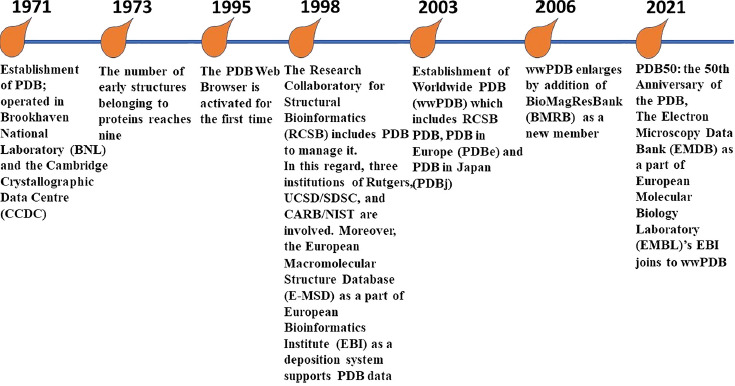
Timeline of historical evolution of Protein Data Bank (PDB)

The Protein Data Bank (PDB) is known as an international virtual data core, which serves as a fundamental information source in association with atomic structures, crystallography and three-dimensional (3D) structures of biomolecules, including nucleic acids and proteins (e.g., enzymes, immunoglycoproteins, adhesins) which are applicable for education and research. In this regard, biotechnology, biopharmaceutics, bioengineering, biomedicine, biology are disciplines that are directly dependent on the use of PDB [1–7]. Indeed, the data and information regarding crystallography and 3D structures of biomolecules released by PDB enable us to have an effective prognostication about the biochemical, biophysical and physicochemical properties comprising affinities and bonds of the related macromolecules and small biomolecules [2, 8–10]. Since 1971, the PDB as the first global open access recourse, which serves invaluable digital data for free. This international public good, supports vital data and information to visualize the biological structures and the related bindings between macro- and small biomolecules. Since 2013, the management of PDB is in accordance with the FAIR (the acronym depicts: Findable, Accessible, Interoperable, Reusable) guiding principles for scientific data [2, 11]. Figure 1 shows the timeline of PDB progression (https://www.rcsb.org/pages/about-us/history) [2, 12–18].

Interestingly, the open access “treasure” of PDB archives and represents several thousands of biomolecules to global users. Atomic and molecular structures of biological molecules together with their complexes (biomolecule-specific ligand(s)) are archived in PDB. Simultaneously, the PDB archive gets bigger and bigger every year. Up to now, the PDB is recognized as a high-managed resource for effective biodata. The FAIR principles are guaranteed via the application of OneDep software system. This software system controls the input structure data receiving by PDB data ecosystem for being validated, standard and biocurated. This process makes the data representing by PDB as findable, accessible, interoperable and reusable [11, 19–21]. Since the establishment of wwPDB [21] in 2003 (Fig. 1) up to now, several biocurators have been recruited by wwPDB centers in different continents such as Asia, Europe and the Americas. A collection of basic sciences and skills comprising enzymology, biophysics, computational chemistry, biochemistry, small molecule crystallography, electron microscopy, macromolecular crystallography and nuclear magnetic resonance (NMR) spectrometry supports the structural biology as the front line aim and goal of the PDB archive [19]. Even during the severe acute respiratory syndrome–related coronavirus (SARS-CoV-2) pandemic era, more than 2000 structures associated with the causative agent of the coronavirus disease (COVID-19) were released and have become accessible for global users for free. A brief collection of PDB deposits is available on SARS-CoV-2 related structures page (https://covid-19.bioreproducibility.org/) [7]. The structural properties of different organisms e.g., COVID-19 released by PDB archives give us this opportunity to find out the spatial conformation of ligands, ligand binding sites, protein-protein interactions and amino acid substitutions regarding different viral proteins. The related data may also be represented by other centers and websites rather than PDB (https://www.rcsb.org/news?year=2020&article=5e74d55d2d410731e9944f52&feature=true), including the COVID-19 Data Portal (https://www.covid19dataportal.org/) and PDBe-KB COVID-19 Data Portal (https://www.ebi.ac.uk/pdbe/covid-19) among others.

Moreover, chemical, functional and energetic characteristics are effective data, which may be gained from PDB to describe the potential capabilities for each individual molecule. These properties belonging to each structure and organisms may support us to determine the potential drug targets for drug design and vaccine preparation [22]. As an important documentary evidences, 210 new molecular entities (NMEs) were discovered and developed during a period of 2010–2016 and then were approved by the US Food and Drug Administration (FDA). The primary 3D structural data and information belonging to all of these NMEs compartments, were first produced and released via PDB archive. The representation of the related structures encouraged pharma companies to finance in drug discovery and development [2, 23]. Due to this fact, the aim of this review article is to show the vital importance of RCSB PDB as a virtual information “treasure” for research in biotechnology.

## METHODS (LITERATURE SEARCH)

The design of the present manuscript is a narrative review, with the aim of critically analyzing and contextualizing the present knowledge and future perspectives on PDB. To formulate the present manuscript, a literature search was performed by the authors in the PubMed/MEDLINE, SCOPUS, EMBASE, and Web of Science databases up to 1st of September, 2021. No restrictions on article type, language or year of publication were set. The authors examined the primary search results and selected papers based on their suitability to be included in this review paper. After the selection of appropriate articles, the reference lists of these papers were also screened for relevant articles. Additionally, in case of some sub-topics of the review, authors also used references from their personal collection, totaling in *n =* 106 references.

## PROTEIN DATA BANK (PDB)

The establishment of PDB in 1971 as an effective global open access resource for biological digital data was initiated by the introduction of only seven structures of proteins; and now at the time of writing this article PDB houses > 182,600 biological macromolecule structures (https://www.rcsb.org/) pertaining to DNAs, proteins, RNAs, these biological molecules complexes with other molecules (e.g., drugs). The foundation of PDB as a unique feature was happened for the first time in the world’s science history. Nowadays, PDB is identified as a remarkable gold standard and a great investment for archiving digital data regarding 3D structures of biological molecules. Therefore, PDB currently is known as an outstanding reference for researchers, trainers and students in the fields of applied and basic sciences associated with biology and biomedicine [23, 24].

For ensuring the highly validation and well-expertized biocurated of archived 3D macromolecular structures in PDB, the International consortium of wwPDB (RCSB PDB [25], PDB in Europe (PDBe) [26], PDB Japan (PDBj) [17] and Biological Magnetic Resonance Data Bank (BMRB) [27, 28]) (Fig. 1) has launched the OneDep software system which is known as a deposition-biocuration-validation tool [29]. These evaluations are achieved through professional expertized processes e.g., 3D cryo-electron microscopy (3DEM), X-ray crystallography and NMR [29]. Indeed, OneDep covers the wwPDB consortium through its unified software tool for deposition, biocuration and validation of the represented archived data associated with macromolecular structures [28]. To promote the validation and the quality of archived structures data in the wwPDB archive, availability of raw experimental data is enforced. OneDep system controls any ambiguity issues associated with experimental data and/or atomic models. This process facilitates the following handling processes for depositors to check and accomplishing any correction regarding a PDB deposition. Further doubtful issues will be rechecked by the manuscript reviewers or via wwPDB biocurators. To reduce the duration of validation process and to convene the validation task forces (VTFs) and effective validation metrics, the wwPDB has recruited a the OneDep software tool (https://deposit.wwpdb.org) for depositors server (https://validate.wwpdb.org/) [29] to check the experimental methodology containing electron microscopy [30], electron crystallography [31], solid-state- and solution NMR [31, 32], neutron diffraction [33], X-Ray diffraction [34, 35], fiber diffraction [24].

## THE ONEDEP SOFTWARE TOOL

The main goal of an open access digital data resource organization like wwPDB is to distribute high-quality data and information with no limitations to its global users. To provide this condition, the PDB archive is supported by strong system to enhance the quality of disseminated data. Today, the PDB archive as a progressive digital data resource encompasses numerous structures which are provided through 3DEM, crystallography and NMR spectroscopy [28]. These progressions are resulting from the successful efforts by the structural biology community. Simultaneously, the PDB archive is responsible for the validity of the released data. Due to this responsibility, since January 2014 the wwPDB employed the OneDep software system to support the atomic 3D structures obtained via crystallography (X-ray). Two years later in January 2016, the OneDep system was recruited for those structures obtained by 3DEM, crystallography (X-ray) and NMR [28]. Interestingly, the advanced OneDep software controls the repositories which are contained of a huge number of experimental data pertaining to crystallography (X-ray), 3DEM and NMR. These professional interoperations ensure the uniqueness of deposited data to assign PDB code. Subsequently, the deposited data get BMRB and Electron Microscopy Data Bank (EMDB) codes. In parallel with this, the employment of advanced OneDep system guarantees the uniformity, quality and accuracy of represented data and information through the wwPDB system [28].

The OneDep software tool is capable to support the most experimental approaches and tools as a single technique or combined ones. Moreover, the OneDep system recognizes and obstructs the defective deposited data; includes the new accepted data for different structures; controls the related data automatically in the process of deposition; checks the pre-validation reports before data deposition, supports the release of the molecular structures under deposition-bio-curation-validation responsibilities in PDB archive and provides a quality service for global depositors in different geographical situation [15, 28, 29]. By conclusion of data deposition through the wwPDB OneDep validation pipeline, a pre-validation report is represented to depositor. The depositor reviews the deposited data to accept or reject pre-validation report. If accepted, the uploaded data undergo for biocuration. The biocurator analyses the accuracy of the obtained data. Accepted data by biocurators enters to the final step as the official validated data. The final validation report will be released by the wwPDB centers [29]. The official validation report issued by wwPDB involves entire quality score for a PDB submission and certain issues. The wwPDB validation reports are accessible through the https://www.wwpdb.org/validation/validation-reports link [15, 28, 29]. The validation report issued by wwPDB is consisted of overall quality at a glance, entry composition, residue-property plot, data and refinement statistics, model quality, fit of model and data [15, 21, 29, 36].

The wwPDB data centers are able to serve their users around the world. The PDBe/UK (www.pdbe.org) supports Europe and Africa, the PDBj/Japan (www.pdbj.org) serves the Middle East and Asia and the RCSB PDB/US (www.rcsb.org) covers the Oceania and Americas [14, 17, 28, 37]. Due to this knowledge, each partner of PDB consortium e.g., PDBe is involved in processes data deposition. In addition, PDBe as a partner participates in archiving and releasing the related data pertaining to molecular structures. In parallel with these activities, the PDBe recruits advanced software tools and systems to serve their users by quality data availability, analyses and visualization. These facilities help the global users from drug discovery researchers to protein engineering scientists to find their target structure(s) much easier and have a fruitful interpretation from the target macromolecular structure(s). All in all, the partners of PDB consortium try to keep data resources in accordance with FAIR guiding principles [11, 15, 37].

## PROTEIN DATA BANK IN EUROPE (PDBE): AN EFFECTIVE PARTNER OF WWPDB

As a partner of PDB consortium, PDBe collaborates with different resources of bioinformatics to enrich its data center. PDBe represents a collection of bioinformatic data through the project of Structure Integration with Function, Taxonomy and Sequence (SIFTS, http://pdbe.org/sifts/) [38]. The SIFTS project provides huge amounts of data pertaining to protein sequences and structures and annotations. This project bridges the core resources of PDBe and the Universal Protein Resource (UniProt) Knowledgebase (UniProtKB, http://uniprot.org) at the European Bioinformatics Institute (EMBL-EBI; http://www.ebi.ac.uk) [38, 39]. A portion of annotation resources which cover the SIFTS project data are consisted of CATH (https://www.cathdb.info) [40], Ensembl (www.ensembl.org) [41], Gene3D (http://gene3d.biochem.ucl.ac.uk/Gene3D/) [40, 42], Gene Ontology Annotation (GO/GOA) (http://www.ebi.ac.uk/GOA) [43], HomoloGene (https://www.ncbi.nlm.nih.gov/homologene) [44], Integrated relational Enzyme database (IntEnz) (http://www.ebi.ac.uk/intenz) [45], Integrative classification of Protein sequences (InterPro) (https://www.ebi.ac.uk/interpro/) [46], Protein families database (Pfam) (http://pfam.xfam.org/) [47], NCBI Taxonomy (https://www.ncbi.nlm.nih.gov/taxonomy/) [48], PubMed (http://www.ncbi.nlm.nih.gov/pubmed) [49] and Structural Classification of Proteins (SCOP) (http://scop.mrc-lmb.cam.ac.uk) [50].

In addition to SIFTS, FunPDBe is another project which supports Protein Data Bank in Europe-Knowledge Base (PDBe-KB) (https://pdbe-kb.org). In another word, the PDBe-KB contains all the data belongs to the projects of SIFTS and FunPDBe. The functional annotations and predictions associated with molecular structures data in the PDB archive are merged and compared through PDBe-KB [51]. Indeed, PDBe-KB supports the enhancement of annotations visibility disseminated by data resources and simultaneously decreases the splitting of annotations [51]. The structural data belonging to PDB are applied via a huge number of scientific software tools and data resources. In parallel with this feature, several numbers of these data resources promote the biological context of macromolecular structures through adding a wide range of effective annotations associated with biophysical and biochemical characteristics relating to data [51]. Due to this knowledge, biomacromolecular tunnels and pores, molecular pockets and channels [52], ligand binding sites [53–55], interactions between biomolecar complexes [56], structural and functional analyses of single nucleotide polymorphisms (SNPs) in biomolecules [57] and proteins catalytic sites [58, 59].

It is important that, several effective centers for bioinformatics e.g., InterPro [46], MobiDB (https://mobidb.org/) [60], PDBsum [61], PDBj [62], Pfam [47], RSCB PDB [63, 64], Reactome (https://reactome.org) [65], SCOP2 [50, 66] and UniProt [67] count on SIFTS as an active resource data to represent fruitful links between PDB consortium and the other biological bioinformatic digital data for serving their global users with up-to-date data and information [38]. The PDBe at the European Molecular Biology Laboratory (EMBL)-European Bioinformatics Institute (EBI) manages PDBe-KB; an activity which is covered by ELIXIR 3DBioInfo community [16, 68, 69]. Molecular recognition of inhibitors, signaling molecules and adaptors and substrates determine the strength of protein functions. Molecular dynamics and the dynamic characteristics of protein molecules are directly involved in spatial configuration and folding and unfolding activities of proteins. In this regard, a mass of software tools and systems has been designed and made [70–74].

The annotations pertaining to structural and functional data associated with proteins represent an effective activity in the field of protein engineering (e.g., antibodies and enzymes). Due to this fact, the canonical structures were identified in spatial configurations of antibodies’ 3D structures within their hypervariable domains. Indeed, the pivotal role of biocomputational methods in determination of canonical structures in 3D structures belonging to immunoglobulin molecules led to influential progression in predictive procedures through the bioinformatic and computational tools and techniques to obtain effective and accurate structural data in antibodies and other proteins. The effective and strong employment of bioinformatic and biocomputational procedures and methodologies in protein engineering resulted in development and progression in biotechnology through the establishment of a significant number of biotechnological companies to represent influent clinical procedures, tools and methodologies for advanced research fields [68, 75, 76].

ELIXIR encompasses a wide range of platforms which is able to support different digital data centers around Europe. The PDBe and InterPro – as the core digital resources of ELIXIR – are linked to other important annotation and structure prediction resources including CATH-Gene3D [42], FUGUE [77], GenTHREADER [78], PHYRE [79], SUPERFAMILY [80] and SWISS-MODEL [81]. Moreover, since 2018 BRENDA enzyme data base (https://www.brenda-enzymes.org) is known as the ELIXIR core data resource (https://elixir-europe.org/platforms/data/core-data-resources), too [82, 83]. BRENDA as a continuous curated system releases effective and reliable data, updated categorization of enzymes and simultaneously involves new identified enzymes. BRENDA shares new and high-quality data to support the needs of global users in the fields of biotechnology, systems biology, pharmaceutics, and medicine [82]. The core data resource of BRENDA belongs to German Network for Bioinformatics Infrastructure (de.NBI (https://www.denbi.de/)) which is covered by the German Node of ELIXIR [82, 84].

The availability, 3D visualization and structural analyses of macromalecules constitute the core of structural biology and structural bioinformatics. Hence, the recruitment of Mol*Viewer as a part of the Mol* open-source project supports the development of a common library and tools for web-based molecular visualization, graphics and analyses. This software tool covers services for the structural biology and structural bioinformatics to feed international PDB consortium [68, 73, 85].

## THE RESEARCH COLLABORATORY FOR STRUCTURAL BIOINFORMATICS PROTEIN DATA BANK (RCSB PDB)

The RCSB PDB – as the US Data Center of wwPDB – serves several thousands of American and Oceanian depositors in Americas and Oceania continents. The US Data Center of serves its millions of global users with a huge number of structural data relating to macromolecules for free, all the disseminated data via wwPDB and in particular RCSB PDB are unlimited and free of charge. It is estimated that more than 660 *k* of RCSB PDB users are students, researchers and educators (from different fields involving bioengineering, biomedicine, biotechnology and fundamental biology) who utilize PDB101 center service (www.PDB101.RCSB.org). Since 2019, the portal of RCSB PDB web has been equipped with modern software tools a systems for an easy search and availability through a full Boolean operator logic [64].

**Fig. 2.**
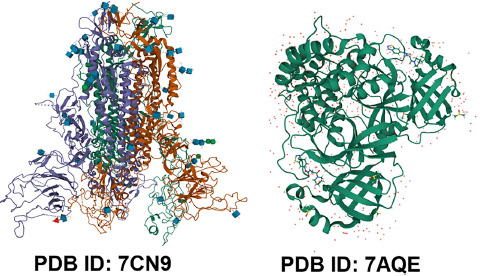
SARS-CoV-2 Spike ectodomain, PDB ID 7CN9; SARS-CoV-2 Main Protease, PDB ID 7AQE

**Fig. 3.**
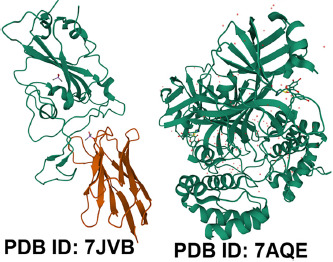
SARS-CoV-2 spike receptor-binding domain (RBD), PDB ID 7JVB; SARS-CoV-2 3CL protease, PDB ID 7DPP

Because of the importance of 3D biostructure data in research and investigation, software tools are developed to manage the related services in the field of bioengineering, biomedicine, biotechnology and fundamental biology [14, 64]. The facilities including search of protein and nucleic acid sequences [86, 87], short sequence motifs in protein and amino acid sequences, protein structure similarities [88], recognition of amino acids constituting binding or catalytic sites and ligands [64]. Due to this information, the 3D biostructure digital data belonging to wwPDB consortium such as RCSB PDB has had pivotal role associated with drug designing, drug discovery targes and vaccines against the COVID-19 pandemic era [2, 23, 89]. At the time of writing this article, by searching the keywords of “‘COVID-19’ drug targets” in RCSB PDB search box you may find 178,740 viral structures (e.g., the SARS-CoV-2 Spike ectodomain, PDB ID 7CN9 [90] (Fig. 2)); SARS-CoV-2 Main Protease, PDB ID 7AQE [91] (Fig. 2); the SARS-CoV-2 spike receptor-binding domain (RBD), PDB ID 7JVB (Fig. 3) [92]; SARS-CoV-2 3CL protease, PDB ID 7DPP [93] (Fig. 3).

RCSB PDB weekly supports PDB structure data through integrating more than 40 external digital biodata resources to refresh and enrich structural views for its global users, many of them are mentioned in the PDBe section [64, 89]. As the RCSB PDB covers US PDB operations, this center receives financial supports from some important institutes including Department of Energy, the National Cancer Institute, the National Institute of Allergy and Infectious Diseases, the National Institute of General Medical Sciences and the National Science Foundation. Moreover, the University of California San Fransisco (UCSF), the State University of New Jersey, Rutgers and the San Diego Supercomputer Center at the University of California San Diego support the human resources and specialists of RCSB PDB [89].

The RCSB PDB as a super-professional data center controls, supports and coordinates the updating process archival data in PDBe and PDBj as the wwPDB international consortium in Europe and Asia, respectively [89]. The RCSB PDB is continuously in progression; the growth of macromolecular structures, small molecule ligands, integral membrane protein structures serves users to apply for biotechnology and the related sciences [89]. Since 2014, the National Institutes of Health (NIH) has started the project of Illuminating the Druggable Genome (IDG); the aim of this project is to detect unknown proteins and to enhance our knowledge regarding those proteins that interact with small molecules. The Target Central Resource Database (TCRD) (http://juniper.health.unm.edu/tcrd/) and Pharos (https://pharos.nih.gov/) are resulted from the IDG project. Both of TCRD and Pharaos as the IDG resources cover the related facilities to have better understanding of undiscovered regions pertaining to human genome [94]. The National Institutes of Health (NIH) Common Fund Data Resources are Pharos [95], Genotype-Tissue Expression (GTEx (https://gtexportal.org)) [96] and the International Mouse Phenotyping Consortium (IMPC (https://www.mousephenotype.org) [97]. The characterized chemical compounds supports a portion of PDB data resource and now are accessible through the wwPDB chemical component dictionary (wwPDB CCD) [98]. Moreover, the DrugBank database (https://www.drugbank.ca) [99], which collaborates with RCSB PDB, disseminates the molecular data and information associated with antibiotics and drugs, drug metabolism, drug pharmacokinetics, drug pharmacodynamics and the mechanism of their activities and the related target molecules. These facilities served by DrugBank provide the researchers to design a wide range of drugs and predict drug metabolites *in silico* [99, 100].

## PROTEIN DATA BANK JAPAN (PDBJ)

The PDBj is the Japanese member of the wwPDB international consortium contributes to biological structures of macromolecules acceptance and annotation together with its other partners such as BMRB, RCSB PDB and PDBe [17, 62]. The PDBj covers the processing and annotation of those depositions received from the Middle East and Asia. All of the partners involving in wwPDB international consortium like PDBj release their updated digital structural data at midnight of Wednesday, every week. The PDBj represents updated databases and remarkable service tools for different research fields of bioinformatics and structural biology [17, 62]. The specific recruited tools in PDBj services consist of PDB mine 2 (which supports the users to search 3D structures with different resolutions and residues and clarifies the PDB metadata) [62], Molmil (a web-based molecular reviewer and graphics program (http://gjbekker.github.io/molmil/)) [62, 101], ProMode-Elastic a normal mode analysis-based database of PDB which is achieved via the program of Elastic-network-model based normal mode analysis (PDBETA) and computes the structures of proteins, DNAs, RNAs and ligands (https://pdbj.org/promode-elastic) [62, 102–104], electrostatic surface of functional-site (eF-site) with virtual reality (VR) technology (a database provides the electrostatic surfaces in association protein functional site (http://www.pdbj.org/eF-site/) [62, 105] and Omakage search (a web-based service to find out the global shape similarities in association with 3DEM or atomic model of biological macromolecules and the related assemblies in EMDB and PDB (https://pdbj.org/omokage) and Gaussian mixture model fitting (Gmfit) program [62, 106].

## CONCLUSIONS

Even since the advent of molecular biology technologies and crystallography, it has been widely recognized that knowledge pertaining to the structures of biologically-relevant macromolecules hold valuable and critical information for chemistry, biology and various branches of medicine. However, since the beginning of the 21^st^ century, the interest in atomic structures, three-dimensional (3D) structures of biomolecules and various molecular interaction studies have received substantial interest, both from researchers in basic science, from pharmaceutical and/or biotechnology companies, and people involved in clinical medicine. Although substantial information in this field is scattered in the literature (both in freely-available and subscription-only sources), there are few relevant, comprehensive and freely available global sources in this field. The Worldwide Protein Data Bank (wwPDB) – and its affiliates – is one of these sources, providing reliable, curated and easily accessible data and tools to visualize biological structures and the interaction between biomolecules on the micro- and macromolecular scale, which may be relevant to all users of the biomedical sciences. The present paper aimed to surmise the main aspects, branches and advantages of using the wwPDB during research and the development for novel pharmaceutical and biotechnological products. As demonstrated by the SARS-CoV-2 pandemic, rapidly reliable and accessible biological data for microbiology, immunology, vaccinology, and drug development are critical to address many healthcare-related challenges that are facing humanity. As a consequence, the importance of databases such as wwPDB has been further validated in recent times, with the expectation that the number of scientists interested in the utilization of Protein Data Bank’s resources will increase substantially in the coming years.
